# Use of attribute association error probability estimates to evaluate quality of medical record geocodes

**DOI:** 10.1186/s12942-015-0019-3

**Published:** 2015-09-15

**Authors:** Christian A. Klaus, Luis E. Carrasco, Daniel W. Goldberg, Kevin A. Henry, Recinda L. Sherman

**Affiliations:** North Carolina Central Cancer Registry, Raleigh, NC USA; North Carolina Center for Geographic Information and Analysis, Raleigh, NC USA; Department of Geography, Texas A&M University, College Station, TX USA; Department of Computer Science & Engineering, Texas A&M University, College Station, TX USA; Department of Geography and Urban Studies, Temple University, Philadelphia, PA USA; North American Association of Central Cancer Registries, Springfield, IL USA

**Keywords:** Record linkage, Health informatics, Biostatistics, Geocoding, Probability, Attribute association, Attribute association error probability, Attribute association hierarchy

## Abstract

**Background:**

The utility of patient attributes associated with the spatiotemporal analysis of medical records lies not just in their values but also the strength of association between them. Estimating the extent to which a hierarchy of conditional probability exists between patient attribute associations such as patient identifying fields, patient and date of diagnosis, and patient and address at diagnosis is fundamental to estimating the strength of association between patient and geocode, and patient and enumeration area. We propose a hierarchy for the attribute associations within medical records that enable spatiotemporal relationships. We also present a set of metrics that store attribute association error probability (AAEP), to estimate error probability for all attribute associations upon which certainty in a patient geocode depends.

**Methods:**

A series of experiments were undertaken to understand how error estimation could be operationalized within health data and what levels of AAEP in real data reveal themselves using these methods. Specifically, the goals of this evaluation were to (1) assess if the concept of our error assessment techniques could be implemented by a population-based cancer registry; (2) apply the techniques to real data from a large health data agency and characterize the observed levels of AAEP; and (3) demonstrate how detected AAEP might impact spatiotemporal health research.

**Results:**

We present an evaluation of AAEP metrics generated for cancer cases in a North Carolina county. We show examples of how we estimated AAEP for selected attribute associations and circumstances. We demonstrate the distribution of AAEP in our case sample across attribute associations, and demonstrate ways in which disease registry specific operations influence the prevalence of AAEP estimates for specific attribute associations.

**Conclusions:**

The effort to detect and store estimates of AAEP is worthwhile because of the increase in confidence fostered by the attribute association level approach to the assessment of uncertainty in patient geocodes, relative to existing geocoding related uncertainty metrics.

## Introduction

Person, place, and time are the three fundamental axes of epidemiology. Most disease surveillance systems now geocode patient data. This, along with advances in geographic analysis software, has led to a revived focus on place in public health research that utilizes secondary data, e.g. from cancer registries. Spatiotemporal analysis relies on spatially enabled data created through data linking and is a valuable tool for epidemiologists, but the utility of this approach depends on the accuracy of the spatial data. Research has shown that geocoding error impacts significantly on the association of enumeration areas with addresses of individuals [1, 2] and subsequently on the stability of incidence rates [3, 4]. Despite this, most epidemiological and cancer research does not currently attempt to describe, account for, or mitigate geocoding accuracy error, or estimate its potential impact on study conclusions [5]. There is an emerging field of literature on the influence of geocoding related data quality on spatiotemporal epidemiologic analysis results [6, 7]. However the impact of uncertainty in person, time and place attributes, that lead to a geocode, is often overlooked.

Person, place and time are identified not with single attributes in a medical record, rather by attribute associations. Attribute associations commonly used to identify person, place and time are described in Tables 1 and 2 (hereafter we use attribute association 1, 2, etc. to refer to categories of attribute associations described in Tables 1 and 2). Thus a patient can be identified with names, date of birth and government issued identifier for most medical records. Estimates of attribute association error probability (AAEP) can be introduced to a medical record during record linkage such as address geocoding and enumeration area assignment.

**Table 1:** Person, place and time attribute associations in patient medical records

Descriptive epidemiology concept	Attribute association description	Core attributes from chronic disease record	Questions relevant to probability of error in attribute association	Association
Person	Patient identifying fields	Patient names, date of birth, government issued ID	What is the probability that the correct patient was not identified?	1
Time	Patient: date of diagnosis	Patient, date of diagnosis, diagnostic confirmation	What is the probability that the correct date of diagnosis was not identified?	2
Place	Patient: address at diagnosis	Patient, date of diagnosis, address, postal code, and postal locality or city	1. What is the probability that the correct address of patient primary residence was not identified? 2. What is the probability that the chosen address does not match to one and only one address in the universe of known addresses?	3

**Table 2:** Patient geocode and enumeration area attribute associations in patient medical records

Attribute association description	Core attributes from chronic disease record	Questions relevant to probability of error in attribute association	Association
Patient: geocode	Patient identifying fields, date of diagnosis, address, postal code, postal locality and geocode	What is the probability that the wrong set of coordinates (and by extension, residence) was chosen during geocoding for the patient?	4
Patient: enumeration area (EA) feature	Patient identifying fields, date of diagnosis, address, postal code, postal locality, geocode, county, sub county enumeration area	What is the probability that the wrong enumeration area was assigned to the patient?	5

It is important for researchers to quantify the AAEP in their data to help guide research design and provide context for result interpretation. But when using secondary datasets, like cancer registry data, the researcher does not have access to the original medical records. So generally, AAEP would have to be generated at the original data source and communicated to researchers.

AAEP would add to the utility of existing metrics for recording geocoding related uncertainty in chronic disease registry data and research. In this paper, we identify a set of AAEP metrics for attribute associations associated with geocoding patient location that could be useful in informing epidemiologic research design and interpreting research results. We review existing data quality metrics that pertain to attribute associations in Table 2, and provide disease registry specific approaches to estimate error in attribute associations within a single medical record. Finally, we use examples from a North Carolina county as a demonstrative case study.

## Background

**Fig. 1:**
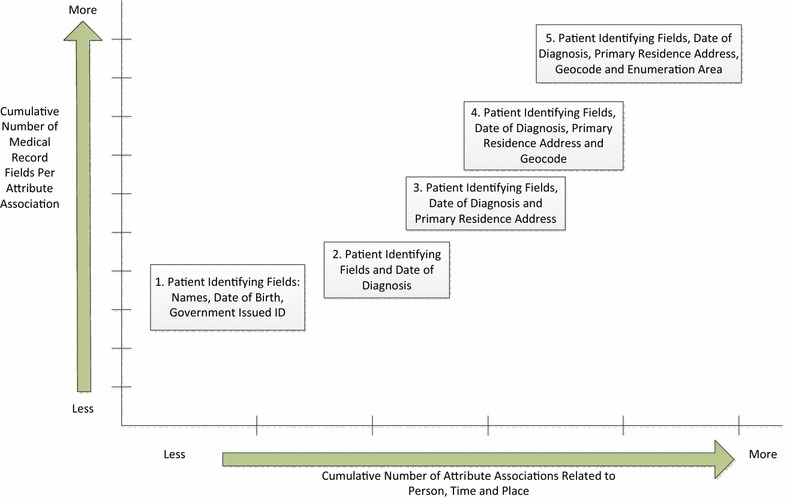
Illustration of hierarchical nature of attribute associations related to geocoding of cancer cases

**Fig. 2:**
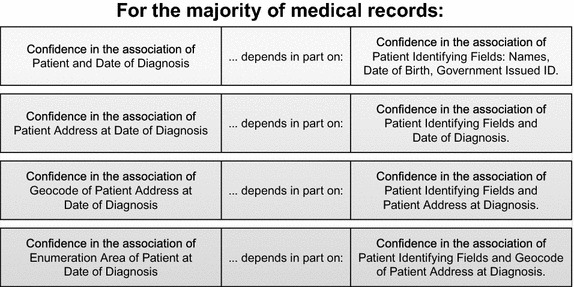
Summary of hierarchical nature of attribute associations related to geocoding of cancer cases

For the majority of medical records, confidence of place is derived in part from confidence of person and time (diagnosis date) in a hierarchical manner as illustrated in Figs. 1 and 2. Confidence in patient geocode is derived in part from all of the above. Record linkage to external datasets is used to detect AAEP, and thereby confirm or contradict conditional probability between attribute associations in a cumulative manner to determine for which attribute associations this hierarchy holds, for a given record. For most cancer records, successful record linkage is leveraged to identify and remove any AAEP for associations 1–2, and then confirm or contradict AAEP in associations 3–5 using geocoding methods that survey data indicate are employed at cancer registries [4, 8]. Approximately half of cancer cases reported to a cancer registry are linked to existing cases and the hierarchy can be confirmed or contradicted through that linkage. For unlinked cases, the hierarchy is presumed based on prior linkages further up the cancer reporting data stream (e.g., at clinics and hospitals) or else information taken directly from the patient. In this way patient attributes in these associations are assumed to store not only their own values but also the attribute association hierarchy through those associations.

Record linkage can also introduce AAEP and thereby bring the hierarchy into doubt. Often due to a lack of options, researchers frequently use addresses geocoded against reference data that have positional and/or attribute error [9–14], in some cases significant enough that it causes incorrect enumeration area (EA) associations [13, 14]. In this way geocoding related error introduces potential bias to epidemiologic studies [3–5, 15–18].

The hierarchy reflects the fact that one linkage can enable another, so the confidence in one attribute association can depend on others, with a common example being the linkage of patient demographic data for the purpose of deriving a street address. Manual patient address correction efforts undertaken by disease registries have shed important light on several factors which influence the occurrence and amount of patient-address AAEP in medical records. For instance in the US, the US Social Security Death Index (SSDI) is widely considered a trusted source of correct information for government issued identifiers. Attempting to mitigate patient address error has led to the use of government sponsored individual centric databases in the US and Australia as a means of updating or verifying street address by linking to patient demographic fields [4, 19]. Examples of these are Birth and Death Records. Others include government maintained Driver’s License and Voter Registration datasets, whose address history for a patient may not be comprehensive to all addresses that a patient lived at, and commercially available databases (such as Lexis Nexis Accurint) that aim to contain a comprehensive address history [20, 21].

Consolidation of patient case-level records across medical records can also play a strong role in whether the hierarchy holds for a given consolidated case. Case-level information is submitted to population-based cancer registries by treating and diagnosing facilities after a patient has been seen by a provider. Because a patient may visit many facilities, the (often many) medical records thus received must be consolidated into a (single) case-level record [22], a process that inevitably introduces some error, such as when a patient address at diagnosis must be chosen from multiple addresses. Multiple addresses occur due to documentation error (e.g., spelling mistakes) or change in residency over time. Error in patient demographic attributes may stem from directly from the patient, or from intermediary providers that processed the patient’s data, or from linking with other source information like mortality files or pathology databases. Regardless of their source, such errors can impact epidemiologic analysis [23].

Another challenge to this hierarchy in patient medical records is competing record linkage and consolidation objectives from contributors to the cancer reporting data stream that process the same records. Obtaining and maintaining an accurate diagnosis address is a priority of disease registries but the priority for medical facilities is to maintain a current address for billing purposes. This discordance can be quantified in error rates of association 3 (patient-diagnosis date-address).

## Motivation

Errors in address reporting are non-random, most significantly impacting medical records of minorities in terms of race, ethnicity, socio-economic standing, and those who reside in rural areas [5, 13]. These issues present significant hurdles for researchers and policy-makers who seek to understand the drivers of disease, the factors which influence uptake in treatments, or the reasons why disparate health outcomes occur between communities.

The identification and removal of geocoding-related error has been the focus of numerous research undertakings presented in the spatial health literature throughout the years. Research has consistently found that, although highly beneficial, manual attempts to identify and correct geocode-related errors represent significant staffing resource investment [24]. While it is possible to manually review the postal address and geocode of every case processed by a disease registry, in practice the effort (and cost) required are deemed to outweigh the potential benefits. Generally a relatively small percentage of cases are manually edited to enable geocoding of their addresses at disease registries.

All of the above has confirmed that within the non-random geocoding related patient data error, patterns exist in the distribution, magnitude, and presence of errors, and that the latter may be skewed towards what can be cost effectively detected. These patterns occur (1) in space—specific regions across a city, county, state, etc. will have different distributions of error; (2) in report source—specific sources of medical records, i.e., different reporting facilities such as hospitals, and government maintained databases of individuals will have different distributions of postal address error associated with a patient; and (3) in person—specific types of patients will have higher propensities toward postal address errors than others. The ability to differentiate types 2 and 3 requires extra effort at the disease registry and thus these are analyzed as a single cause in this paper. We note further that differentiating AAEP introduced into medical records by at least some patients, from that introduced by the treating/diagnosing facility processing the patient’s data, or the disease registry that consolidates them, might ultimately require estimating AAEP at the time and place of patient intake.

Currently there are no metrics for researchers who utilize data from disease registries that can account for AAEP to inform the appropriateness of data for specific research questions or designs, for the attribute associations outlined in Tables 1 and 2. Disease registries typically use intra-record field consistency checks for a subset of all patient and tumor attribute associations submitted to them by hospitals, through a pass-fail method that relies on domain constraints. Place attributes include relatively less domain-constrained text fields for which consistency checks based on limited domains are not as effective. This is partly due to the sheer number of alphanumeric address aliases used in both medical records and geographic reference data that cannot be resolved by commonly used address standardization and parsing algorithms. Furthermore, pass–fail does not allow for communication of a range of confidence in a given attribute association. In recognition of this, international medical data standards-setting organizations for cancer registries utilize two data quality items that attempt to standardize confidence levels associated with disease registry records for specific attribute associations. GIS Coordinate Quality Code (GIS-CQC) and the Census Tract Certainty Code (CTC) [25] are detailed in Appendix 1. These attempt to describe, in a qualitative manner with mostly ordinal rankings, common data quality issues of just associations 4 and 5 in Table 2.

These data quality items provide precedent to the concept of AAEP but are primarily applicable to US and Canadian addresses. There is also significant precedent for the concept and applicability of AAEP, especially for association 4 in Table 2, to addresses in many other parts of the world. We note in particular the *geocoding certainty indicator* (GCI) and accompanying conceptual schema of address components developed by Davis and Fonseca, for use with addresses in Brazil and many other countries [26]. Furthermore, methods of estimating AAEP can be considered by drawing on an international body of data mining literature describing methods and frameworks for modeling and representing hierarchical attribute association uncertainty [27–31]. Examples of these include probabilistic XML and Dempster–Shafer theory, and similar frameworks employed to represent and develop probabilities of information uncertainty both within an extensible medical record and by merging different extensible records together [27–29].

## Experimental evaluation

We conceptualize assessing error in the probability of association of two or more attributes, on a scale from 0 to 1, where null indicates AAEP was not assessed, 0 indicates error assessed but not detected, 1 indicates error determined and not correctable, and the values in between indicate error probability. Having a probability range enables the development of rules whereby the degree of AAEP might be differentiated from one medical record to another for a given attribute association.

## Methods

For a disease registry, aspects of record linkage design and execution such as standardization, parsing, record linkage algorithm, and matching threshold play a role in record linkage success and thus a potential role in estimation of AAEP. AAEP could also conceivably be assessed by (1) comparing associations in patient data, via record linkage, to corresponding ones in external data that contain an approximation of the universe of known and valid attribute associations; (2) relative frequency analyses of attribute combinations in such external data, for instance first, middle and last names; (3) quantification of probability that attributes exist in same time line in external data, for instance address location at time of diagnosis; (4) quantification of probability that a match is one to one; (5) quantification of impact to AAEP estimates of record linkage metadata such as the number of data sets linked, their vintages, accuracy and, particularly for geocoding, precision of features matched to and spatial concordance thereof, concordance of input and matched attributes, strength of linkage and linkage date; and (6) the intuition and experience of the record linkage analyst to assess error in both sides of a data linkage. Assessing AAEP based on this or a larger list is beyond the scope of this paper and represents topics for future research. Nevertheless the list hints at the variety of assumptions that might require Bayesian computation to be incorporated into an AAEP estimate, or else might be packaged as metadata to accompany an AAEP estimate, and ways that AAEP might be detected, given those assumptions.

Exactly how AAEP estimates might be generated depends on the extent to which AAEP can be detected, which depends to some degree on the availability of external data sets to link patient data against and record linkage objectives and constraints such as time available for record linkage. A list of data sets commonly linked to by disease registries to link patient data against is detailed in Table 3. The choice of datasets to link to is also dependent upon who is assessing AAEP, as different organizations have access to different external datasets for record linkage and different record linkage objectives that guide the choice of those datasets, even though they may work with the same data (patient medical records) as in the case of hospitals and disease registries.

**Table 3:** Centralized databases that disease registry cases are commonly linked to

Number	Name	Description	Purpose of Linkage
1	Admission records (admission table in CCR database)	One or more admission records are consolidated to generate a single tumor level record	Record consolidation
2	Medicaid	Medicaid claims data, generally for a rolling 5 year period	Casefinding; patient attribute confirm/update
3	Hospital discharge	Discharge sheets submitted by hospitals to state health authorities	Casefinding
4	Rapid case ascertainment	Databases of smaller subsets of patients who meet criteria for enrolling in research studies	Casefinding; patient attribute confirm/update
5	Dept. of Motor Vehicles Driver’s License Data	US state database storing the demographic and other attributes of drivers	Patient attribute confirm/update
6	Board of Elections’ Voter Registration Data (BOE-VR)	US state or county database storing demographic and other attributes of voters	Patient attribute confirm/update
7	National Death Index	List of deceased persons, aggregated across US states	Patient attribute confirm/update
8	State Death Registry	List of deceased persons, aggregated across counties in a state	Casefinding; patient attribute/update
9	Social Security Death Index(SSDI)	List of deceased persons, aggregated across counties in a state, with US Social Security Numbers	Casefinding; patient attribute confirm/update
10	Government Postal Address Database	Used to clean addresses, or diagnose address problems	Patient attribute confirm/update
11	GIS Street Centerlines	Digital linework corresponding to center of US streets, with address and postal code attributes. Used for navigation and emergency response	Geocoding
12	GIS parcels	Digital parcel polygons corresponding to property ownership, with some site address attributes. Used for tax assessment	Geocoding
13	GIS address points	Digital points, generally corresponding to a primary residence within a parcel. Used for navigation and emergency response	Geocoding
14	Census enumeration area polygons	Digital polygons, approximating delineation of census enumeration areas (EA). Used for spatial overlay to assign EA	Assignment of EA to patient record
15	Census enumeration area table	Table that can be joined to using geocoding reference identifiers, to assign enumeration area	Assignment of EA to patient record

For the sake of demonstration, we selected methods of AAEP estimate generation that accommodate standard disease registry operational constraints in alignment with typical cancer registry record linkage objectives. These are generally to enable enumeration area based and patient residence based epidemiologic reporting and research. Thus, we wanted to use AAEP estimates to notify researchers of the ability to differentiate a patient geocode (and by extension, residence) from other geocodes or patient enumeration area from other enumeration areas.

## The ESTREAA hierarchy

AAEP is tied closely to the hierarchy of attribute associations in Figs. 1 and 2 because of the potential for propagation of AAEP from one attribute association to another. As there is conceivably more than one hierarchy of attribute associations within medical records, we decided to term this the entity spatiotemporal relationship enabling attribute association (ESTREAA) hierarchy of conditional probability between attribute associations. If attribute associations in external data to which patient attribute associations are linked differ from the patient attributes (so that 0 < AAEP > 1), then patient attribute associations are inconsistent with each other, and the hierarchy may not hold for that association and larger ones. If on the other hand the attribute associations in external data are the same as in the patient data, the patient attributes are consistent with each other and the hierarchy is presumed to hold (AAEP = 0) for that association and larger ones. For cancer records, information on record linkage based verification of attribute associations may not be available for associations 1–3 at the time AAEP is assigned. By convention, if the AAEP estimate for associations 1–3 is null, we must presume that the ESTREAA hierarchy holds for those associations.

## Categorizing the ESTREAA hierarchy for a given medical record

Specific attribute associations enable specific relationships to external data at case level. Examples of these for epidemiology include relationships based on spatial proximity via association 4, those based on association with enumeration area based data via association 5, and those based on temporal proximity to environmental exposure event data via association 3. The importance of correctly communicating limitations to these relationships to data users is apparent in currently employed geocoding data quality items (GIS Coordinate Quality and Census Tract Certainty for US and Canadian addresses in Appendix 1) if only indirectly. A corresponding data quality item specific to attribute association has the advantage of linking attribute associations to the relationships they enable. This is key to enabling researchers to successfully subset cases based on quantified limitations to those relationships at attribute association level.

Toward this end, the ESTREAA hierarchy is a logical framework from which to create categories that describe unique combinations of AAEP and how they limit those relationships for a given medical record, and account for propagation of AAEP across associations. When AAEP propagates from one association to another, for example rendering address at diagnosis uncertain when the date of diagnosis is uncertain, then Bayesian computation can be used to estimate AAEP across those associations, subsequent to the estimation of AAEP for a single association through record linkage alone. By recording whether AAEP was propagated or not, categories can be designed to communicate whether AAEP in associations 4 and 5 is due to the patient medical record (so that for example AAEP in associations 1–3 propagates into 4 and 5), geographic reference data, or both. This can be of utility to researchers for study design, replication, and interpretation.

**Fig. 3:**
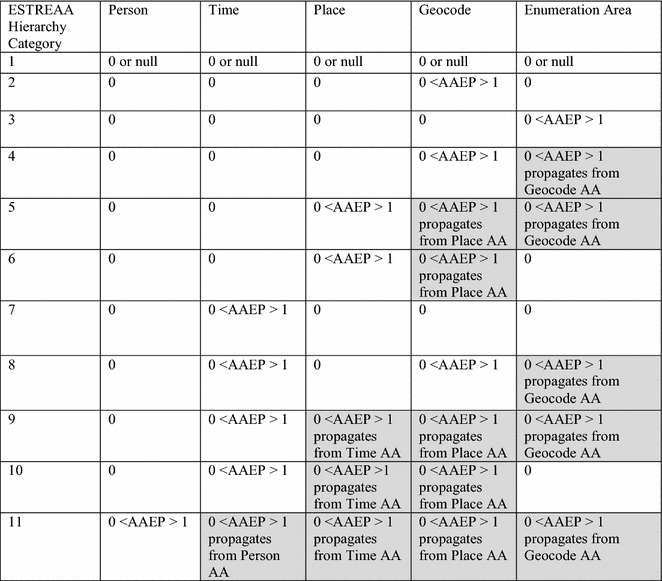
Entity spatiotemporal relationship enabling attribute association (ESTREAA) hierarchy categories selected for attribute associations in Tables 1 and 2. *White background* indicates AAEP evaluation based on record linkage. *Grey* indicates AAEP propagating from a smaller to larger attribute association. *AA* Attribute Association

For purposes of demonstration we found it necessary to limit the conceivably large number of categories, so we selected 11 mutually exclusive categories drawing from our experience working with cancer cases (Fig. 3 and Table 4). The categories we use here are not exhaustive of all unique combinations of attribute associations based on whether AAEP > 0. Through their mutual exclusivity such categories can effectively standardize use cases that might be encountered across disease registries. The circumstance column in Table 5 details some use cases of AAEP estimation encountered in our case study sample. We used these to develop ESTREAA hierarchy categories in Fig. 3 and Table 4, and to identify patterns by which AAEP is propagated across attribute associations. Thus, AAEP in association 1 (patient) always propagates into associations 2–5, and AAEP in association 3 (place) always propagates into association 4 (geocode). By contrast, AAEP may or may not propagate from association 2 (time) into 3 (place), and from 4 (geocode) into 5 (enumeration area).

**Table 4:** Descriptions and examples of entity spatiotemporal relationship enabling attribute association (ESTREAA) hierarchy categories

ESTREAA hierarchy category	Description/example	#(%) of cases in case study sample
1	No AAEP detected (AAEP = 0), or AAEP is null, in all associations	12,791 (95.69 %)
2	AAEP detected in association 4, but contained spatially to enumeration area of interest. An example is a case with a missing house number, on a street wholly contained within the enumeration area of interest	27 (0.20 %)
3	For these cases associations 1–4 are free of AAEP, but association 5 is not. Examples include cases where a county boundary intersects a parcel (found in eastern seaboard states of US), or for which there is disagreement about what enumeration area a parcel belongs to, between local and national government agencies, for example, between US counties and the US Census Bureau, regarding the correct county	0
4	AAEP in association 4 causes AAEP in association 5. An example is a case whose address matches to postal code only. The postal code area overlaps with the enumeration area but is not coincident with it	185 (1.38 %)
5	AAEP in association 3 propagates into associations 4 and 5. An example is a case with a ‘Multimatch’ Address: patient address contains error in more than one address component and matches to more than one candidate based on which component is edited. Another example: patient owns more than one residence and primary residence cannot be determined	74 (0.57 %)
6	AAEP in association 3 propagates into association 4, but uncertainty is contained spatially in one enumeration area of interest so association 5 remains free of AAEP	0
7	AAEP in patient date of diagnosis (association 2) does not impact the choice of address at diagnosis (association 3). Examples include cases for which date of diagnosis is unknown (death certificate only cases) but patient has never changed residence in his/her lifetime. Another example is a clinically diagnosed case for which year of diagnosis is known and month and day is uncertain, but does not affect the choice of patient address at diagnosis	13 (0.097 %)
8	Patient date of diagnosis (association 2) is uncertain, but this does not affect association 3. Association 4 has AAEP based on record linkage, but, similar to category 2, it does not propagate into association 5	0
9	Patient date of diagnosis (association 2) is uncertain, and this affects the choice of address at diagnosis (association 3), which affects the confidence in the geocode, which affects the confidence in the enumeration area. Examples include death certificate only cases where date of diagnosis is unknown	276 (2.06 %)
10	Cases where patient is positively identified, but all other associations have AAEP except association 5. An example is a patient whose date of diagnosis is uncertain, which propagates AAEP into associations 3 and 4, however the uncertainty is contained spatially to enumeration area of interest, and so association 5 is free of AAEP	0
11	Cases where patient is not positively identified (association 1), and AAEP from that association propagates into all other associations	0

**Table 5:** How ESTREAA hierarchy category might be assigned, and AAEP might be estimated for selected circumstances

Example	Attribute association	Circumstance	How AAEP was estimated
1	1. Patient identifying fields	Patient lacks government issued ID and address, and patient names and date of birth match to three individuals in external data	The patient could be three different persons matched to in various sources. The AAEP is calculated as 1 − (1/3) or 0.666
2	2. Patient-date of diagnosis	Patient diagnosis year known, but month and day unknown	One day out of 365 is chosen, thus the probability of choosing the wrong day is 1/365. AAEP = 1 − (1/365) = 0.997
3	3. Patient: date of diagnosis-address	Patient address is missing house number	Patient address matches to the address features^a^ of 20 residences on one street. AAEP = 1 − (1/20), or 0.95
4	3. Patient: date of diagnosis-address	Patient address is missing prefix direction. To confirm that address is valid, it is matched to USPS ZIP + 4 database	Patient address matches to 2 addresses in USPS ZIP + 4 database. AAEP = 1 − (1/2) or 0.5
5	3. Patient: date of diagnosis-address	Error suspected in more than one component of patient address (‘multimatch’ address). Patient address can be matched to 12 different address features in geographic reference data depending on which address component(s) are edited	AAEP = 1 − (1/12) or 0.916
6	3. Patient: date of diagnosis-address	Address at diagnosis cannot be geocoded. Patient address history unknown or incomplete. Patient address identified via linkage to external source on patient name and date of birth, and used to match to geographic reference data with one to one match. Date of diagnosis was not spanned by duration of address validity in external data source	AAEP estimated at 0.25 based on best available information about error rate of external data source
7	3. Patient: date of diagnosis-address	Patient has PO Box address. Patient address history unknown or incomplete. Patient names and PO Box address match to owner names and mailing addresses of 4 parcels, whose sale dates precede the date of diagnosis	There are 4 possible addresses and only one is chosen. AAEP = 1 − (1/4) = 0.75
8	3. Patient: date of diagnosis-address	Patient year of diagnosis known. Patient day and month of diagnosis is unknown. Patient address history for year of diagnosis is known. During that time patient lived at 3 addresses in sequence for 0.4, 0.1, and 0.5 % of the year; the first address is chosen	AAEP = 1 − 0.4 or 0.6 %
9	4. Patient: date of diagnosis-address-geocode	Patient address matches to a street in geographic reference data with 21 address features that are missing house numbers	Patient address matches to address features of 21 residences on one street. AAEP = 1 − (1/21), or 0.952
10	4. Patient-date of diagnosis-address-geocode	Patient street address could not be matched to street level geographic reference data. Patient postal code matched to postal code area centroid	Postal code encompasses 13,500 address features. AAEP = 1 − (1/13,500) = 0.999
11	5. Patient-date of diagnosis-address-geocode-enumeration area	Patient address lacks a house number. Street to which patient address is geocoded is contained within 1 enumeration area	Because all potential matches are contained within the chosen enumeration area, AAEP = 0
12	5. Patient-date of diagnosis-address-geocode-enumeration area	Patient address lacks a house number; there are 70 address feature matching candidates. The area of uncertainty that contains the potential matches spans 2 enumeration areas. These contain 20 and 50 candidate address features; the latter enumeration area is chosen	AAEP = 1 − (50/70) = 0.285
13	5. Patient-date of diagnosis-address-geocode-enumeration area	Patient street address could not be matched to street level geographic reference data. Patient postal code matched to postal code area centroid. Postal code area spans 4 enumeration areas, which contain 2160, 1620, 1620 and 6750 address features respectively; the latter enumeration area is chosen	Postal code encompasses 12,150 address features. AAEP is 1 − (6750/12,150) = 0.444
14	5. Patient-date of diagnosis-address-geocode-enumeration area	Both patient address and postal code are unmatched in geographic reference data. County centroid is assigned as geocode	AAEP is 1 − (1/395,909 address features in county) or 0.999

^a^Emergency dispatch address features as published by county or city data authors

For the categories to be applicable to many countries we have included only one general enumeration area column in Fig. 3. In practical application the categories would have to be extended by substituting actual enumeration areas in place of of the single enumeration area association in Fig. 3 and Table 4. Further, the availability of comprehensive patient address histories plays a decisive role in assigning ESTREAA categories to a medical record. For our study we did not have access to these data but note their importance nonetheless. We have designed the ESTREAA categories around the current convention of geolocation of patients to their address at time of diagnosis. Both the hierarchy and its constituent attribute associations could be modified if the convention were to change, requiring for example AAEP estimates for all addresses in a comprehensive patient address history.

## General approaches to estimating AAEP

We used three approaches to estimate AAEP for specific associations, all based on specific limitations to record linkage that incurs AAEP. These are convention, AAEP substitution across attribute associations, and estimation of AAEP based upon record linkage matching candidates. Selected examples of how AAEP might be estimated are detailed in Table 5, and these examples guided estimation of AAEP for our case study.

The first approach is used when record linkage has failed for a patient medical record. Thus in some cases when AAEP is detected it cannot be resolved or reliably estimated through record linkage. Disease registry conventions for determining residency at time of diagnosis can be used to estimate AAEP when information that might inform a probability is lacking. For example by current convention homeless patients are geocoded to their diagnosing or treating medical facility [32]. The AAEP could arguably be 1 (error) since the patient could not reside at the hospital except for inpatient stay(s). An argument can also be made that since the residential history of the patient is unknown and it’s possible the patient lived on hospital grounds for a time, another value might be more appropriate. But in either case there was no additional information to guide a decision, so that a convention for assignment of AAEP in this case would be ideal. We did not have any of these cases in our sample.

A second approach is to apply substitution of AAEP across attribute associations. In our sample there are cases for which record linkage reduces or eliminates AAEP for one attribute association and introduces or increases it in another. For example, if a street address was derived for a patient from Driver’s License data that was based on dates after the patient’s diagnosis, then association 3 (patient and address) was assigned an AAEP of 0.25 (example 6 in Table 5). We felt this represents the risk that the patient address at time of diagnosis as identified in the Driver’s License database is incorrect, even if evidence for choosing that value for a penalty is not necessarily available for a given case. For these cases, the hierarchy may not hold for associations 4 and 5, although street level geocode and enumeration area are assigned.

Using substitution for the above example, the Driver’s License address can be subsequently removed from the medical record, and the geographic uncertainty increased by geocoding to postal code centroid. AAEP estimates thus enable data users the ability to substitute AAEP across associations by increasing AAEP in one association in order to decrease it in another, if data quality assumptions about data to which cases are linked, change.

With the third approach, when record linkage to external data indicates two or more matching candidates for a given association, then an AAEP estimate is based on the number of those candidates. A chosen value (i.e., of patient, diagnosis date, patient address, geocode) of 1 is divided by the number of candidates that, on account of uncertainty, could have been chosen with as much justification as the actual choice. For attribute associations 4 and 5 AAEP estimates may be strongly determined by whether matching candidates are spatially interspersed with nonmatching candidates or contained spatially to a delimited area containing only matching candidates.

Uncertainty of match between patient address and geographic reference data address can create a single delimited area of uncertainty for their geocodes that vary in size in proportion to the uncertainty involved, as described by Goldberg and Goldberg et al. [4, 33]. Nearly all (99.9 %) of North Carolina cancer cases are geocoded to US county level or lower. The spatial uncertainty associated with a county centroid would be the geographic boundary of the county. The next level of spatial resolution would be the postal code associated with the address. If correct, the output geocode could be assumed to fall within the geographic bounds (or estimation thereof) of the postal code. Following this, the uncertainty of a geocode could be bounded to the convex hull of all streets within a postal code containing the correct street name assuming the street name are correct. Following this, the geocode could be bounded to the convex hull of all streets having the correct name in conjunction with other street attributes aside from the house number including the street type (“Street”, “Drive”, “Avenue”, etc.) as well as the street pre- and post-directionals (“North”, “South”, etc.) assuming each are correct; if incorrect, the convex hull of all streets having the same name would be reasonable (if assumed to be correct). Finally, using the house number attribute (“123”), it would be possible to bound the spatial error of the output to a single matching street segment assuming it and all other address attributes are correct; if incorrect, the convex hull of all streets with the appropriate name and other street attributes would be the best that could be achieved.

We selected matching candidate geocodes to auto-generate convex hulls. In some cases convex hulls spanned features that were not matching candidates or did not span relevant features that were missing address information and on account of that were not considered geocoding candidates. In these situations we interactively delineated areas of uncertainty to span the desired features representing the residential domain of uncertainty for a given case. We could not always rely on the number of residential address features in a given area to compute AAEP, for example when we suspect a newer subdivision is not reflected in the most recent address feature data. We estimated AAEP based on neighboring densities in the interim. We stored the polygonal areas of uncertainty to enable the revision of AAEP with the update of geographic reference data.

We kept AAEP stemming from patient medical records accounted for separately from that stemming from geographic reference data. This enables keeping AAEP current to the update cycles of each. It also enables future identification of causation of AAEP in cases for which neither source can be currently pinpointed as the cause of error, to be assigned as updates are made to either patient medical record or geographic reference data.

## Considerations in estimating AAEP for specific attribute associations

In the following sections we discuss specific aspects of AAEP estimation for each attribute association in Tables 1 and 2.

### Attribute association 1: patient identification

We examined our cases for deceased patients with missing or invalid government issued ID (Social Security Number), that matched to more than one record (person) in the US Social Security Death Index (SSDI) on names and date of birth. Such a patient would have an uncertain identity as SSDI is published by the US Government and is considered the authoritative source on identifying attributes of deceased US citizens. In our sample we did not find any cases with AAEP for this association.

### Attribute association 2: verification of date of diagnosis

AAEP for this association can be estimated on the small percentage, 2.2 % of NC Central Cancer Registry (NC CCR) cases, that were submitted to disease registries by death certificate notification only, because the possibility that the date of diagnosis is the same as date of death is remote and the possibility that the person died before developing cancer is zero (cases identified by autopsy only are coded to the patients date of death). We also estimated it for a small percentage of cases not reported by death certificate only, where year of diagnosis was known but not month and day. For these (typically clinically diagnosed) cases we estimated the probability that the date of diagnosis is incorrect is 1/365 (AAEP = 99.7 %).

For generating AAEP estimates for Tables 1 and 2, verification of date of diagnosis was important only in that it was needed to identify patient address at date of diagnosis.

### Attribute association 3: patient address at date of diagnosis

In the epidemiologic context, association 3 equates to place, and by extension (for chronic disease medical records), primary residence. Primary residence was the criteria by which association 3 was tested in our case study. Generating a probability that an address was not a patient’s primary residence address at time of diagnosis was undertaken only for a subset of cases interactively geocoded. In other words, the precondition to patient address research was that the reported address failed to batch geocode. For these cases, some patient addresses were replaced with addresses from the driver’s license database, to enable match at residence level. Otherwise, the medical facility address was considered more correct than a driver’s license address. Batch geocoding false positive matches were searched for subsequent to batch geocoding using text string similarity measures of input and matched addresses, and none were found.

There are two types of error in this association 3 that must be considered, and in disease registries they are typically assessed at the same time. They are: (1) what is the probability that the wrong address of primary residence at time of diagnosis was assigned to a patient; (2) what is the probability that the patient primary residence address does not match to one and only one address in the universe of known addresses. In most cases, answering the first question depends on whether the second question can be answered. Without comprehensive patient address histories, or in dealing with patients without known addresses, it is often impossible to disentangle these two questions, and so we employed one AAEP estimate for association 3 for both types of error. By contrast if aforementioned comprehensive patient address histories are available, so that patient addresses are known for given time spans, these questions can be answered separately for the majority of patients. If AAEP were to be estimated for both questions then the AAEP estimate for association 3 might be computed with Bayes formula to reconcile the two probabilities.

### Attribute association 4: patient-geocode

In contrast to AAEP estimates for association 3, that for associations 4 and 5 are evaluated once a geocoding match has been made, and reflect any AAEP contributed by attribute associations in both patient medical record and geographic reference data or geographic reference data alone, but not patient medical record alone.

Associations 4 is unique among patient attribute associations in that spatial uncertainty is commonly substituted (so that AAEP is incurred) to enable successful record linkage. For association 4 if the combination of address and postal code is unknown or cannot be geocoded, postal locality or city is substituted by geocoding to that centroid; if postal locality centroid or city centroid cannot be geocoded, then county centroid is substituted.

AAEP for the association of patient residence (place) with street centerline interpolated geocodes might arguably be 1 because the patient has a probability of living at any point in a parcel, but no probability of living in the middle of a street. However it’s not particularly meaningful to epidemiologic inquiry to assign an AAEP of 1 to association 4 for addresses geocoded to a centerline, because in general they never had a chance to be 0 in the first place, since centerlines by design don’t generally intersect parcels. For that reason we chose to not assign 1 to association 4 for those cases in our study sample. More useful is a probability communicating how well a centerline geocode approximates the parcel of residence. We could not address how AAEP might be meaningfully estimated when association 4 involved these geocodes, in this paper, given the scope of the topic. We plan to address the issue in future research.

### Attribute association 5: patient-enumeration area

For this study the enumeration area of interest was the 2010 US census tract. For association 5, patient-enumeration area AAEP was detected using one method for interactively geocoded cases, and another for batch geocoded cases. For the former, AAEP for association 5 was based on aforementioned areas of uncertainty in geocode precision detected during interactive geocoding. For batch geocoded cases, AAEP was detected using patient geocode enumeration area discordance, when a patient address matched to more than one set of geographic reference data. Cases whose geocodes matched to more than one set of reference data comprised ~75 % of study sample. We found the distribution of association 5 AAEP for different reference data pairs for these cases to be generally binary, where one set of reference data was always correct, and the other incorrect, for most EA discordant reference data pairs. This prompted us to choose the geocodes of the reference data judged to be accurate within the EA, and so remove AAEP for association 5 for those cases.

## Data

This case study used 13,366 consolidated NC CCR cancer cases within Wake County diagnosed between 2008 and 2012. The geographic reference data for cadastral and emergency response functions of this county are tightly integrated. Therefore, the subset of reference data features with AAEP was expected to be low for county maintained geographic reference data, and data sets derived from them, such as those maintained by the US Census Bureau or vendors. The effect of our choice of county was to minimize AAEP introduced by geographic reference data relative to the expected contribution from a more rural county had we chosen one of those instead.

## Data linked to

Our estimation of AAEP was driven by the processes of address validation and geocoding. After being validated against the USPS ZIP + 4 database with Satori Bulk Mailer, the cancer cases were geocoded in automated batch-geocoding mode using ESRI ArcGIS 10.1 (criteria: match score 100, no ties accepted). This system uses a probabilistic geocoding algorithm, based in part on the Felligi-Sunter record linkage model and the default weighting schemes of 3 types of ESRI locators: US postal codes, street centerlines and address points (ESRI, Redlands, CA, USA). The same data sources utilized in the batch match process for geocoding were used for interactive geocoding. These included address points for most NC counties (2009, 2011), parcels for all NC counties (2010), and street centerlines for all NC counties (2007, 2010). Both NC Driver’s License Data (2010) and NC parcels were used to research addresses for patients whose addresses were found to have data quality problems that prevented geocoding to street level or better. Patients were matched to parcels by parcel owner names, date of diagnosis and parcel date of sale, and/or mailing address.

There were 483 cases which initially failed batch geocoding and were manually reviewed by NC CCR staff trained in geocoding. These ranged from ~4 to ~8 % of all cases, on a reporting facility basis. We used a .NET/SQL Server ESRI ArcMap Add-In application that, subsequent to assignment of geocode to a given medical record, enabled assignment of AAEP estimates to a case, assignment of ESTREAA hierarchy category to a case, recording of geocoding metadata such as number of candidates for an address and their candidate scores, match score, any enumeration area discordance, a convex hull or user delineated area of uncertainty if applicable, and comments particular to a given attribute association. We generated or recorded AAEP estimates for 21 attribute associations that are mostly component associations of those in Tables 1 and 2.

## Results

### ESTREAA hierarchy categories for case study cases: Table 4

Counts of our case study cases as they were assigned ESTREAA hierarchy categories are summarized in Table 4.

### Address validation and geocoding success in study sample cases: Table 6

Address validation and geocoding success, and percentages of cases geocoded through batch and interactive methods are summarized in Table 6 for the study sample of cases.

**Table 6:** Address validation and geocoding success for cases in wake county, 2008–2012

Case count	Address validation success^a^	Batch geocoded	Interactively geocoded	Geocoding success rate (street level)	Geocoding success rate (postal code level)	Geocoding success rate (county level only)
13,366	87.2 %	95.8 %	3.6 %	96.9 %	1.4 %	0.5 %

^a^Percentage of addresses matched to the USPS ZIP + 4 database prior to geocoding

### Enumeration area (2010 Census Tract) discordance by geographic reference data pair: Table 7

Only rarely was AAEP triggered by error in the geocoding reference data. Percentages of cases with census tract discordance are summarized, for the subset of cases whose patient addresses geocoded to more than one set of geographic reference data, by geographic reference data pair. The percentage is calculated as the number of patient addresses with EA discordant geocodes to the total number of cases geocoded to street level or better. In Wake county there is very little EA discordance among the reference data for geocoded cases, which helps to confirm that the contribution of reference data to AAEP is marginal for the study sample.

**Table 7:** Percentages of selected patient records (2008–2012) with enumeration area discordance for patient address, by geographic reference data set used for batch geocoding, in Wake County

	City or county maintained parcel centroids and/or address points, 2009	Street centerline (state of NC maintained) 2007	TIGER street centerlines 2010	City or county maintained Parcel centroids and/or address points, 2011
City or county maintained parcel centroids and/or address points, 2009		<0.1 %	0.1 %	<0.1 %
Street centerline (state of NC maintained) 2007	<0.1 %		0.6 %	<0.1 %
TIGER street centerlines 2010	0.1 %	0.6 %		<0.1 %
City or county maintained parcel centroids and/or address points, 2011	<0.1 %	<0.1 %	<0.1 %	

AAEP in associations 3 and 4 may be labor intensive to detect. By contrast, for geocoded records, enumeration area based domain checks can easily comprehensively detect AAEP in association 5, as either column domain constraints or else topologically via spatial overlay.

### Percentages of cases with AAEP by medical facility and attribute association: Table 8

Table 8 illustrates the distribution of AAEP for a subset of cases in our sample data. Cases with AAEP in any of the five attribute associations are summarized for four selected medical facilities that submitted them to NC CCR. Slight differences are apparent in the distribution of these cases for these facilities. Although the percentages are low, their magnitude and distribution remains unknown without AAEP estimates. AAEP estimates in this way can foster confidence that AAEP rates are within acceptable ranges for a given medical facility. Were they not, then central registries can choose to intervene with training or other measures to lower the rates.

**Table 8:** Percentages of cases with AAEP in Wake County, 2008–2012, for Four NC medical facilities

Medical Facility	Patient identifying fields (%)	Patient-diagnosis date (%)	Patient address at diagnosis date (%)	Patient address at diagnosis date-geocode (%)	Patient-enumeration area at diagnosis date (US Census Tract, 2010 Census) (%)
1	0	0.9	<0.1	1.08	0.9
2	0	0.38	<0.1	2.57	2.18
3	0	0.71	0	0.71	0.57
4	0	0.25	0.13	1.16	0.58

The percentages of cases with patient-enumeration area (association 5) AAEP are less than the corresponding percentage of patient-geocode (association 4) AAEP for all medical facilities. This reflects the fact that for many cases, patient geocode AAEP was not sufficient to cause AAEP in association 5.

The relatively scarce amount of AAEP in patient identifying fields reflects the strong effort at both reporting medical facilities and disease registries to remove any uncertainty from associations 1 and 2. In contrast to these, removing AAEP for association 3 was less a priority during cancer case consolidation, and is thus detected in a relatively greater percentage of cases (during geocoding), and for all submitting facilities.

## Discussion

By convention, error is not tolerated within cancer record data fields in attribute associations in Tables 1 and 2. For domain constrained fields in an attribute association, error (AAEP = 1) is corrected upon discovery. By contrast, error probability (0 < AAEP > 1) is tolerated and may not be corrected, or even identified. In general we do not have confidence in assigning AAEP = 1 to associations that include fields not domain constrained, because these are linked to a variety of datasets to confirm or contradict them, all of which contain some error of their own. The overall effect of this convention is to exclude conditions under which AAEP would need to be estimated as 1, in other words, error discovered which cannot be corrected or replaced with error probability. We only assigned a value of 1 as a temporary way to record that error was discovered, and that further record linkage was needed to replace it with error probability, and AAEP for all associations were reduced to a value less than 1 before being used for analysis.

The last two columns in Table 8 document the impact of AAEP on association 4 (patient-geocode) and association 5 (patient-enumeration area), respectively. The former is of importance when designing studies that need to control for AAEP to evaluate data variability across primary residences, e.g. the variation of radon test kit results by residence in a neighborhood. The latter, when designing studies that rely on the linkage of cases with enumeration area based information such as census data items.

Examination of AAEP estimates for a sample dataset and the spatial distribution of cases with AAEP within it affords researchers a variety of options with regard to study design. Cases in a cancer study sample may already be heavily filtered on several dimensions, for example on molecular markers, tumor attributes, years of diagnosis, stage, etc., so that the problem of not enough sample, and small numbers impeding stable enumeration area based rates, weighs heavily on study design.

Disease registries and researchers can use AAEP estimates to model the filtering of cases with AAEP from a study sample against analytic power requirements to inform a study design that optimizes for both constraints and thereby confidence in study conclusions. Such filtering may bias sample selection and would best be used with caution.

If sample size is too small for filtering, researchers have the option of weighting cases by AAEP estimate, in proportion to their error probabilty, as model inputs, for example with cluster detection software. Other options include (a) increasing the scale of enumeration area used for analysis to span convex hulls of uncertainty and thereby negate or minimize their effects on analysis and (b) requesting an alternative data set with a more favorable AAEP distribution and/or changing the research objectives to fit the limitations of the data.

AAEP estimates can potentially be shared among researchers without violating health care confidentiality statutes. Thus AAEP estimate distributions (similar to Table 8) for study sample data can be published by researchers in table format to communicate AAEP in their datasets to authors of research with similar objectives to provide an attribute association specific gauge of comparability between the studies’ data sets.

The findings of the experimental evaluation have relevance to the use of geocoded medical records in the following additional ways:

- Confidence in spatiotemporal relationships in which an entity (patient in this study) takes part can be estimated to the degree that AAEP can be estimated, for all attribute associations that enable and/or modify those relationships.
- ESTREAA hierarchy categories can be designed to communicate whether and how AAEP is propagated across attribute associations in a given medical record.
- General approaches to AAEP estimation include conventions, substitution of AAEP across attribute associations, and use of record linkage matching candidates.
- Follow back from the central registries to medical facilities with relatively high AAEP rates can prompt additional review processes at the facilities and potentially reduce sources and magnitude of AAEP error.
- AAEP estimates can be developed that meet specific analysis requirements of other secondary data, like patient insurance claims datasets and other types of electronic medical records.
- Generating and applying standardized AAEP estimates may become an integral component of research that employs health informatics and biostatistics.

## Conclusion

Through an examination of the shortcomings of existing approaches to computing and reporting error for geocoded disease registry cases we find a need for probability based, attribute association specific error metrics. We have provided a hierarchy-based theoretical framework within which these estimates can be generated which follows from the core methods and data sources which are presently used internationally to geocode medical and public health records. This framework enables an accounting of error probability in all attribute associations upon which certainty in a patient geocode depends. Our approach complements existing geocode related error metrics and has been shown to be computable with some additional effort on the part of disease registry staff. The methods we used to assess AAEP in the NC CCR data demonstrate an approach that other disease registries could employ. From the researcher perspective, AAEP estimates are a quantifiable measure generated by the stewards of secondary datasets. Although AAEP may not be detected in all attribute associations in which it exists—owing to constraints under which medical record producers operate—the effort to detect and store it is worthwhile. The distribution of AAEP across and within cases can be summarized to inform study design and interpretation, and provide researchers a common method to compare geocoding data quality in their datasets to other researchers with similar data and research objectives.

## Appendix 1

### Census tract certainty

#### Description

Code indicating basis of assignment of census tract for an individual record. Helpful in identifying cases tracted from incomplete information or P.O. Box. This item is not coded by the hospital. Central registry staff assign the code.

#### Codes

1: Census tract based on complete and valid street address of residence

2: Census tract based on residence ZIP + 4

3: Census tract based on residence ZIP + 2

4: Census tract based on residence ZIP code only

5: Census tract based on ZIP code of P.O. Box

6: Census tract/BNA based on residence city where city has only one census tract, or based on residence ZIP code where ZIP code has only one census tract

9: Not assigned, geocoding attempted

Blank: Not assigned, geocoding not attempted

### GIS coordinate quality

#### Description

Code indicating the basis of assignment of latitude and longitude coordinates for an individual record from an address. This data item is helpful in identifying cases that were assigned coordinates based on incomplete information, post office boxes, or rural routes. This item is coded at the central registry, not by the reporting facility. Most of the time, this information is provided by geocoding software. Alternatively, a central registry staff member manually assigns the code. Codes are hierarchical, with lower numbers having priority.

#### Rationale

Spatial analysis of cancer data often requires identifying data records with a high degree of geographic precision. Researchers can use this code as a basis for selecting records with a degree of precision that is appropriate to the study.

#### Codes

00 Coordinates derived from local government-maintained address points, which are based on property parcel locations, not interpolation over a street segment‘s address range

01 Coordinates assigned by global positioning system (GPS)

02 Coordinates are match of house number and street, and based on property parcel location

03 Coordinates are match of house number and street, interpolated over the matching street segment‘s address range

04 Coordinates are street intersections

05 Coordinates are at mid-point of street segment (missing or invalid building number)

06 Coordinates are address ZIP code + 4 centroid

07 Coordinates are address ZIP code + 2 centroid

08 Coordinates were obtained manually by looking up a location on a paper or electronic map

09 Coordinates are address 5-digit ZIP code centroid

10 Coordinates are point ZIP code of Post Office Box or Rural Route

11 Coordinates are centroid of address city (when address ZIP code is unknown or invalid, and there are multiple ZIP codes for the city)

12 Coordinates are centroid of county

98 Latitude and longitude are assigned, but coordinate quality is unknown

99 Latitude and longitude are not assigned, but geocoding was attempted; unable to assign coordinates based on available information

Blank GIS coordinate quality not coded

## Abbreviations

AAEP: attribute association error probability
EA: (Census) Enumeration Area
ESTREAA Hierarchy: Entity Spatiotemporal Relationship Enabling Attribute Association Hierarchy
